# Gut microbes involvement in gastrointestinal cancers through redox regulation

**DOI:** 10.1186/s13099-023-00562-z

**Published:** 2023-07-13

**Authors:** Wang Yangyanqiu, Chu Jian, Yang Yuqing, Qu Zhanbo, Han Shuwen

**Affiliations:** 1 Huzhou Central Hospital, Affiliated Central Hospital Huzhou University, No. 1558, Sanhuan North Road, Wuxing District, Huzhou, 313000 Zhejiang Province China; 2 Graduate School of Medical College, Zhejiang University, No. 268 Kaixuan Road, Jianggan District, Hangzhou, 310029 Zhejiang Province China; 3 Zhejiang Chinese Medical University, No. 548 Binwen Road, Binjiang District, Hangzhou, 310053 Zhejiang Province Republic of China; 4 Key Laboratory of Multiomics Research and Clinical Transformation of Digestive Cancer, No. 1558, Sanhuan North Road, Wuxing District, Huzhou, 313000 Zhejiang Province Republic of China

**Keywords:** Gastrointestinal microbes, Gastrointestinal cancers, Redox, Reactive oxygen species, Oxidative stress

## Abstract

Gastrointestinal (GI) cancers are among the most common and lethal cancers worldwide. GI microbes play an important role in the occurrence and development of GI cancers. The common mechanisms by which GI microbes may lead to the occurrence and development of cancer include the instability of the microbial internal environment, secretion of cancer-related metabolites, and destabilization of the GI mucosal barrier. In recent years, many studies have found that the relationship between GI microbes and the development of cancer is closely associated with the GI redox level. Redox instability associated with GI microbes may induce oxidative stress, DNA damage, cumulative gene mutation, protein dysfunction and abnormal lipid metabolism in GI cells. Redox-related metabolites of GI microbes, such as short-chain fatty acids, hydrogen sulfide and nitric oxide, which are involved in cancer, may also influence GI redox levels. This paper reviews the redox reactions of GI cells regulated by microorganisms and their metabolites, as well as redox reactions in the cancer-related GI microbes themselves. This study provides a new perspective for the prevention and treatment of GI cancers.

## Introduction

According to Global Cancer Statistics 2020, gastrointestinal (GI) cancers, including colorectal cancer and gastric cancer, account for approximately one-third of the total global cancer incidence and mortality [1]. The GI tract is estimated to contain over 70% of all microbes in the human body [2]. Arguably, the most important microbial communities in humans can be found in the GI tract. A finely regulated relationship exists within the GI tract, however, microorganisms essential for host health can also contribute to the occurance and development GI cancers [3–5]. For example, *Helicobacter pylori* induces gastric cancer by modulating CagA, the oligotoxin VacA allele and other cytokines that affect the body’s immune system [6, 7]; *Haemophilus ducreyi* induces cell cycle arrest by inducing the tripartite cytolethal distending toxin that creates DNA lesions [8]; *Enterococcus faecalis* mediates colorectal cancer (CRC) by producing genotoxic peroxides that influence the cell cycle and polyploidy precipitation [9, 10]; *Salmonella* promotes CRC by releasing the multifunctional protein AvrA, which inhibits β-catenin degradation, or by altering β-catenin ubiquitination and acetylation levels [11].

The reduction and oxidation (redox) state of the GI tract depends on the balance of antioxidants and oxidants. Common antioxidants in the GI tract are glutathione and superoxide dismutase (SOD) [12–14]. In addition, common oxidants in the GI tract are free radicals, including superoxide (O_2_·−) and hydroxyl (OH·); reactive oxygen, including hypochlorous acid (HOCl) and hydrogen peroxide (H_2_O_2_); nitrogen species, including nitric oxide (NO) and nitrogen dioxide (NO_2_·); and sulfur species, including hydrogen sulfide (H_2_S), persulfides and polysulfides [14–22]. When the redox state is unbalanced due to an increase in oxidants or a decrease in antioxidants, GI cells undergo oxidative stress. Exogenous and endogenous sources of antioxidants and oxidants both have effects on the overall redox state of the GI tract. Xenobiotics, including gut microbes, are important regulators of redox potential in the GI tract. For example, Sannasimuthu A et al. found that mice treated with broad-spectrum antibiotics showed a change in the “redox potential” of their gut environment [23]. Mitochondrial respiratory chain enzymes, including the cytochrome P450 enzyme family and xanthine oxidase in the endoplasmic reticulum, cytoplasm and nuclear membrane, are the main endogenous sources [24–26]. Homeostasis of GI cells is influenced in a variety of ways by the GI redox state, including modulation of signal-transduction pathways, induction of DNA strand breaks and modifications of proteins and lipids [27–29]. Oxidative stress has been implicated in a series of GI diseases, including GI cancers [30].

**Fig. 1:**
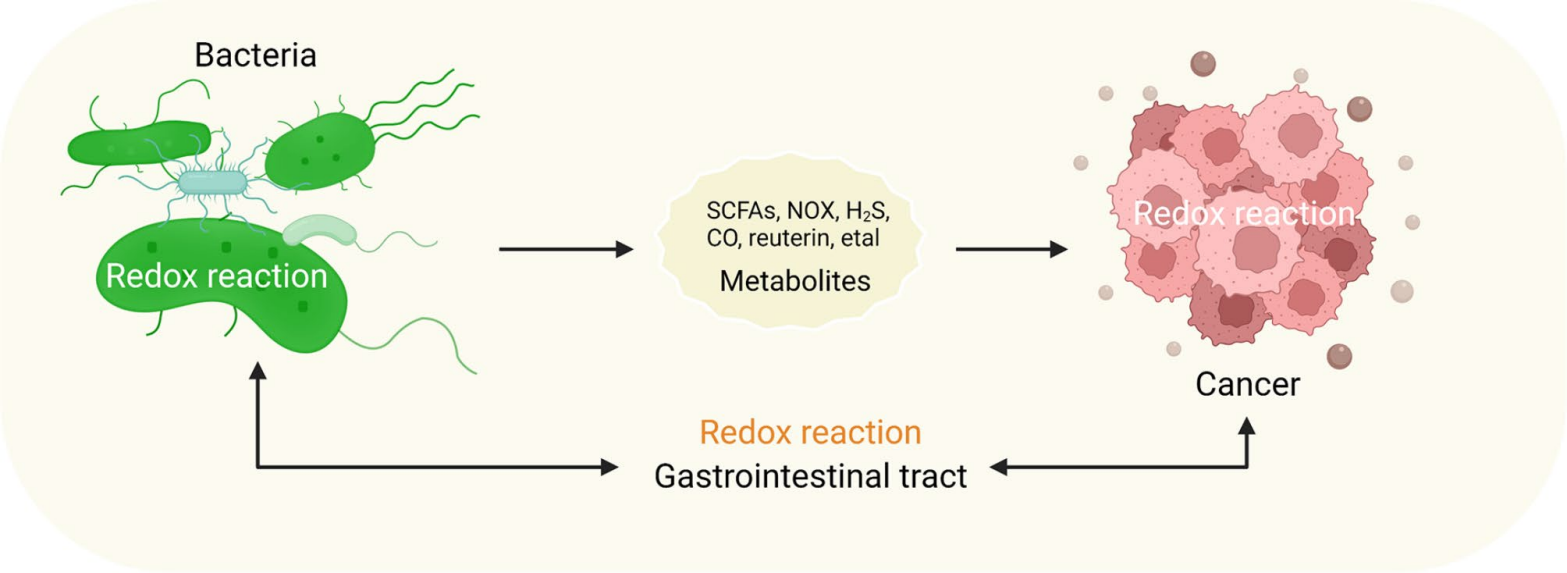
The relationship between bacteria, their metabolites, and cancer involves redox reactions. Bacteria and their metabolites regulate cancer through redox reactions

GI microbes are involved in the occurrence and development of GI cancers and influence the redox level of the GI tract. Understanding the regulatory mode of GI microbes from the redox view may be a guide for the prevention and treatment of GI cancers. Therefore, this paper reviews the redox changes in cancer-related GI microbes and cancer cells affected by GI microbes and their metabolites in detail (Fig. 1).

## Redox reactions in carcinogenic bacteria influence redox balance in the GI tract

**Fig. 2:**
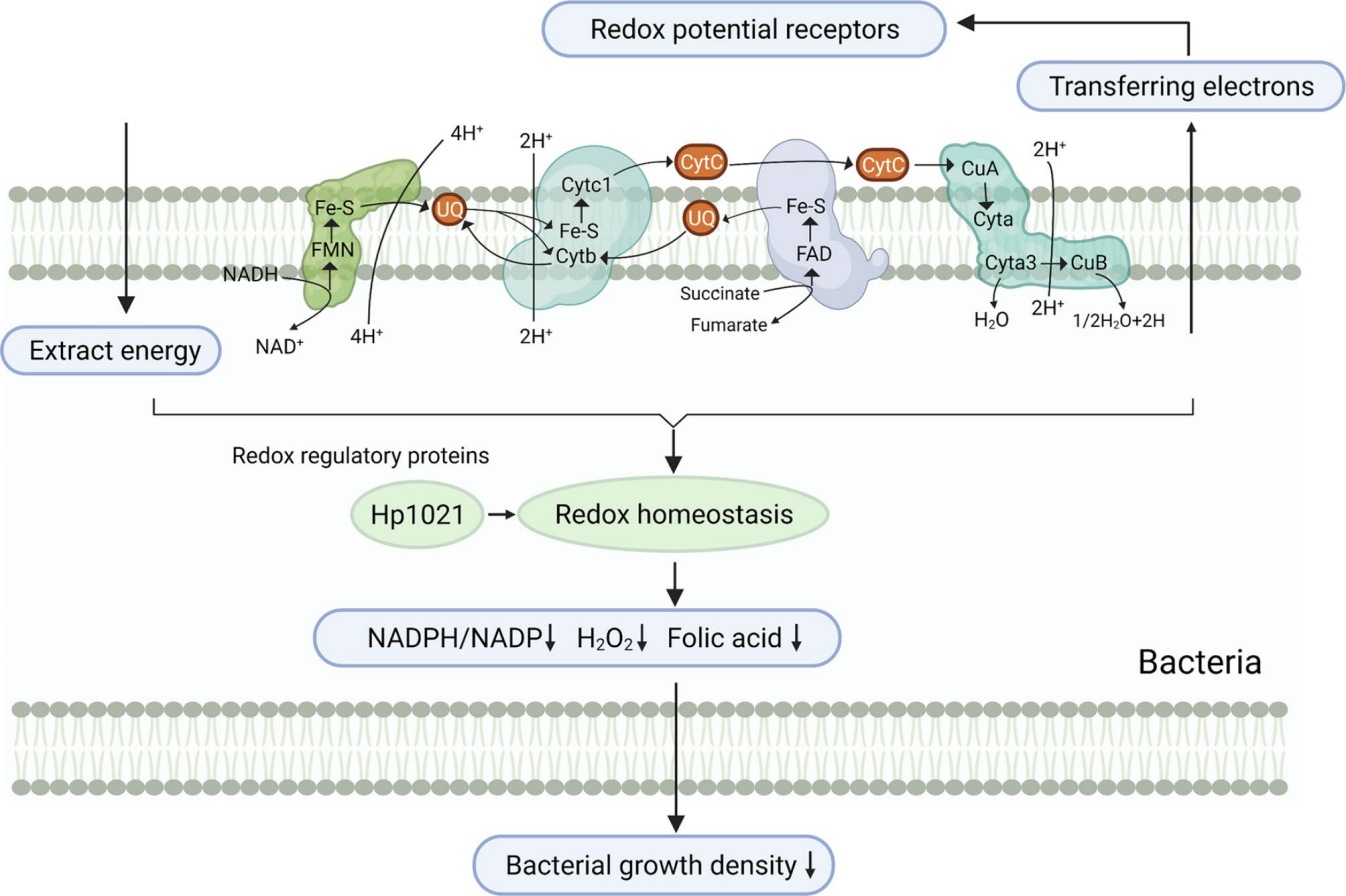
Redox reactions in bacteria regulate the redox level of the gastrointestinal tract. Bacteria obtain the energy needed for growth through the respiratory chain and transfer electrons in vivo and in vitro through the respiratory chain at the same time, thereby regulating the redox level in vivo and in vitro. Redox regulatory proteins such as HP120 exist in bacteria. Oxidative metabolism in bacteria can affect the production of its metabolites, such as folic acid. Reduced production of folic acid contributes to the occurance of cancer. The change in redox level in the gastrointestinal tract caused by bacteria can also affect the growth of other bacteria

GI bacteria are one of the sources of GI redox potential, and there are redox systems, including the respiratory chain, in GI bacteria. Bacteria are prokaryotes, and their respiration mainly occurs on the cell membrane and is catalyzed by enzymes on the cell membrane. Therefore, the cell membrane is the main site of oxidizing agents produced by bacteria. Most oxidants in bacteria are produced by a continuous univalent electron transfer reaction of oxygen molecules catalyzed by enzymes in the respiratory chain (Fig. 2). For example, *Escherichia coli* produces approximately 87% hydrogen peroxide in its respiratory chain [31]. Microbes use a very complex electron transport system to extract energy from the intestinal environment, while the bacterial respiratory cascade ultimately transfers electrons to higher redox potential receptors in the extracellular space. During normal bacterial proliferation, this universal, inducible electron outflow mechanism leads to changes in the redox potential in the GI tract, the extent of which depends on the growth stage, microbial population, and physiology of the organism [32]. The GI redox potential is also related to the intestinal microbial composition. For example, intestinal oxidation status was negatively correlated with the abundance of *Lactobacillus* and *Bifidobacterium* and positively correlated with the abundance of *Escherichia coli* [33].

Carmelini et al. indicated that bacterial cells respond to oxygen stress in the GI tract by slowing growth and regulating the expression of proteins involved in carbohydrate uptake and metabolism, redox homeostasis, the DNA damage response and bacterial movement [34]. *Clostridium butyricum* responds to obvious redox during the growth process by changing from an oxidized state to a reduced state and growing at a slower rate. When *Clostridium butyricum* cells undergo stress, the expression levels of several genes are upregulated, including those associated with pyruvate metabolism, the conversion of acetyl-CoA to acetaldehyde and the stress response [35]. *Helicobacter pylori* is a microaerobic carcinogen that encodes a redox switch protein, Hp1021. The cysteine residues of Hp1021 are sensitive to oxidation in vitro and in vivo, and the DNA-binding activity of Hp1021 to oriC depends on the redox state of the protein. Hp1021 is directly involved in the oxygen-dependent control of the *Helicobacter pylori* feca3 and GLUP genes, which are related to the oxidative stress response [36]. Menadione and H_2_O_2_ have been used to induce oxidative stress in the model intestinal bacterium *Enterococcus durans* (MTCC 3031). Oxidative stress significantly reduced the redox ratio (NADPH/NADP) by 55% (menadione) and 28% (H_2_O_2_). The reduction in the redox ratio caused by oxidative stress decreased bacterial folate synthesis. Reduced folic acid levels can induce colorectal cancer [37]. In addition, oxidative stress significantly reduced bacterial growth density by 61% (menadione) and biological yield by 21% (H_2_O_2_) [38].

## GI bacteria induce oxidant and antioxidant production in cancer cells

Several types of gut bacteria can influence the production of oxidants and antioxidants in gastrointestinal cells [39]. Oxidants produced by gastrointestinal cells drive abnormal cell proliferation and survival by activating signaling pathways such as HIF-1α. Wen J, et al. indicated that *Helicobacter pylori* infection could significantly upregulate the expression of AQP3 and HIF-1α in human gastric epithelial AGS and GES-1 cells and increase ROS levels. *Helicobacter pylori* infection could stimulate the ROS-HIF-1α axis and ROS-HIF-1α-AQP3-ROS ring, promoting the occurrence of gastric cancer [40]. Zhang X et al. reported that *Lactobacillus* stimulated the production of NADPH oxidase-1 (Nox1)-dependent ROS and induced cell proliferation of GI stem cells [41]. Lesiow MK et al. showed that under the stimulation of two fragments of FomA adherin from *F. nucleatum* and its complexes with copper (II) [Cu(II)-Ac-KGHGNG-NH_2_ (1Cu) and Cu(II)-Ac-PTVHNE-NH_2_ (2Cu)], large quantities of hydroxyl radicals were produced inside and outside of colon cancer CT-26 cells. The intense oxidative stress caused by these compounds could trigger a cascade of free radicals in CT-26 cells, leading to substantial lipid peroxidation [42]. Microbes in the GI tract may also increase oxidation levels by reducing antioxidant levels. For example, Nie Seru et al. found that *Clostridium* infection in gastric cancer was negatively correlated with three metabolites, including glutathione, uric acid and pyrophosphate, and confirmed that glutathione levels decreased if the nuclear abundance of *Clostridium* was high. In addition, some microorganisms in the GI tract can reduce high oxidation levels by upregulating the expression of antioxidants in GI cells [43]. In GES-1 cells with acute infection, nuclear *Clostridium* upregulated GSH peroxidase 4 (GPX4) and GSH synthetase (GSS) expression and reduced glutathione (GSH) to oxidized glutathione (GSSG), resulting in the reduction of intracellular ROS levels and increasing the ability of cells to combat self-damage [44]. Wang Yue et al. showed that *Streptococcus thermophilus* CGMCC 7.179, under oxidative stress induced by 2 mm H_2_O_2_, could improve the activities of major antioxidant enzymes (superoxide dismutase, glutathione peroxidase and catalase) in CRC HT-29 cells and protect CRC cells against oxidative stress [45, 46].

## Excess oxidants from the GI tract or GI cells may be involved in the destruction of DNA, proteins and lipids in cancer cells

### Excess oxidants are involved in cancer cell DNA damage

GI symbiotic microbes affect genomic stability in GI cells by overproducing oxidants. *Enterococcus faecalis* and *Bacteroides fragilis* have been found to be associated with colorectal cancer, and *Enterococcus faecalis* and *Bacteroides fragilis* infection are accompanied by excess ROS production [47, 48]. ROS can induce DNA base oxidation (e.g., formation of 8-oxo guanine) and single strand breaks, which can be repaired by base excision/single strand breaks. Both base excision repair and single strand break repair are performed by DNA polymerase, which forms long or short fragments that are ligated under the action of DNA ligase III or DNA ligase I. If single strand breaks and base excision repair/single strand break repair occur simultaneously on opposing strands, DNA double-strand breaks can be generated [49–52]. Wang S et al. found that *Helicobacter pylori* infection could induce gastric cancer cells to activate the NF-κB signaling pathway and upregulate peroxyredoxin2 (PRDX2) expression [53, 54]. ROS can affect replication fork progression through the dissociation of PRDX2 oligomers. PRDX2 can form a replica-related ROS sensor. The ROS sensor is combined with the DNA replication fork accelerator Timeless. The increase in ROS leads to the separation of PRDX2 and Timeless, thus slowing the speed of replication bifurcation [55]. Oxidized bases occurring from ROS activity also present a physical obstacle to replication forks, resulting in the breakdown of replication forks at fragile sites across the genome [56, 57]. Fork breakdown leads to DSBs and ultimately underreplicated or overreplicated DNA, with concomitant genomic instability in cancer cells. Chronically high ROS levels can outpace a host’s DNA repair mechanisms, leading to DNA damage and mutations [58]. At present, ROS have not been found to directly affect the function of DNA double-strand break repair proteins. Break-induced replication has been recently implicated in the repair of replication stress or nuclease-induced DNA double-strand breaks at telomeres [59]. With the clinical implications of interfering with DNA repair pathways becoming apparent, the direct effect of bacteria-inducing ROS on DNA repair proteins requires more investigation.

### Excess oxidants are involved in the redox modifications of proteins in cancer cells

Multiple studies have suggested that ROS participate in a series of biological processes, such as metabolism and oxidative stress defense. The common mode of action of ROS is to react with the nucleophilic mercaptan group (-- SH) in a specific protein cysteine, which results in a series of oxidative posttranslational modifications (oxiPTM). Mercaptan can be oxidized to S-sulfinic acid (–-SOH) by ROS. S-sulfinic acid has high activity and can be continuously converted into more stable forms, including S-sulfinic acid (–-SO_2_H) and disulfide (–SS-). The cysteine regulation of reversible oxidized proteins is an important mechanism that changes the function of proteins after translation [60, 61]. The metabolic enzymes in many metabolic processes, including glycolysis, the tricarboxylic acid cycle, lipid metabolism, energy metabolism and amino acid metabolism, could be regulated by redox modification [62–64]. *Escherichia coli* may induce metabolic reprogramming changes in heterogeneous cancer cell populations by modulating glycolytic pathway-inducer genes and oxidative phosphorylation-related genes (such as pyruvate dehydrogenase kinase 1, pyruvate dehydrogenase phosphatase, and malonyl-CoA-acyl carrier protein transacylase). After metabolic reprogramming, the migration and self-renewal capacity of cancer cells can be enhanced [65]. ROS produced by bacteria and GI cells can oxidize cysteines 45 and 392 of PDHK2 (Cys45 and Cys392), thereby inhibiting PDHK2 activity, dephosphorylating PDH, and ultimately promoting the tricarboxylic acid cycle [66]. As a result, cancer cells can continuously obtain the energy they need to survive.

### Excess oxidants are involved in the redox modifications of lipids in cancer cells

Lipids are one of the main structural and functional components of biological tissue, especially the cell membrane. After lipid peroxidation, GI cell membrane fluidity and cell membrane properties are changed [67, 68]. Excess peroxidation of free radicals can lead to iron overload [69]. Membrane-associated phospholipid peroxide glutathione peroxidase (GPX4) protects against iron-associated cell death by preventing the accumulation of lipid peroxide, demonstrating the damage of excess oxidants to lipids on GI cancer cell membranes [70]. Oxidants on the GI cell membrane regulate signaling receptors on the cell membrane and various downstream signaling pathways, including cytokines such as tumor necrosis factor (TNF) and growth factors such as epidermal growth factor receptor (EGFR)[71]. Alterations in signaling receptors on the membrane of GI cancer cells can affect the function of cancer cells.

**Fig. 3:**
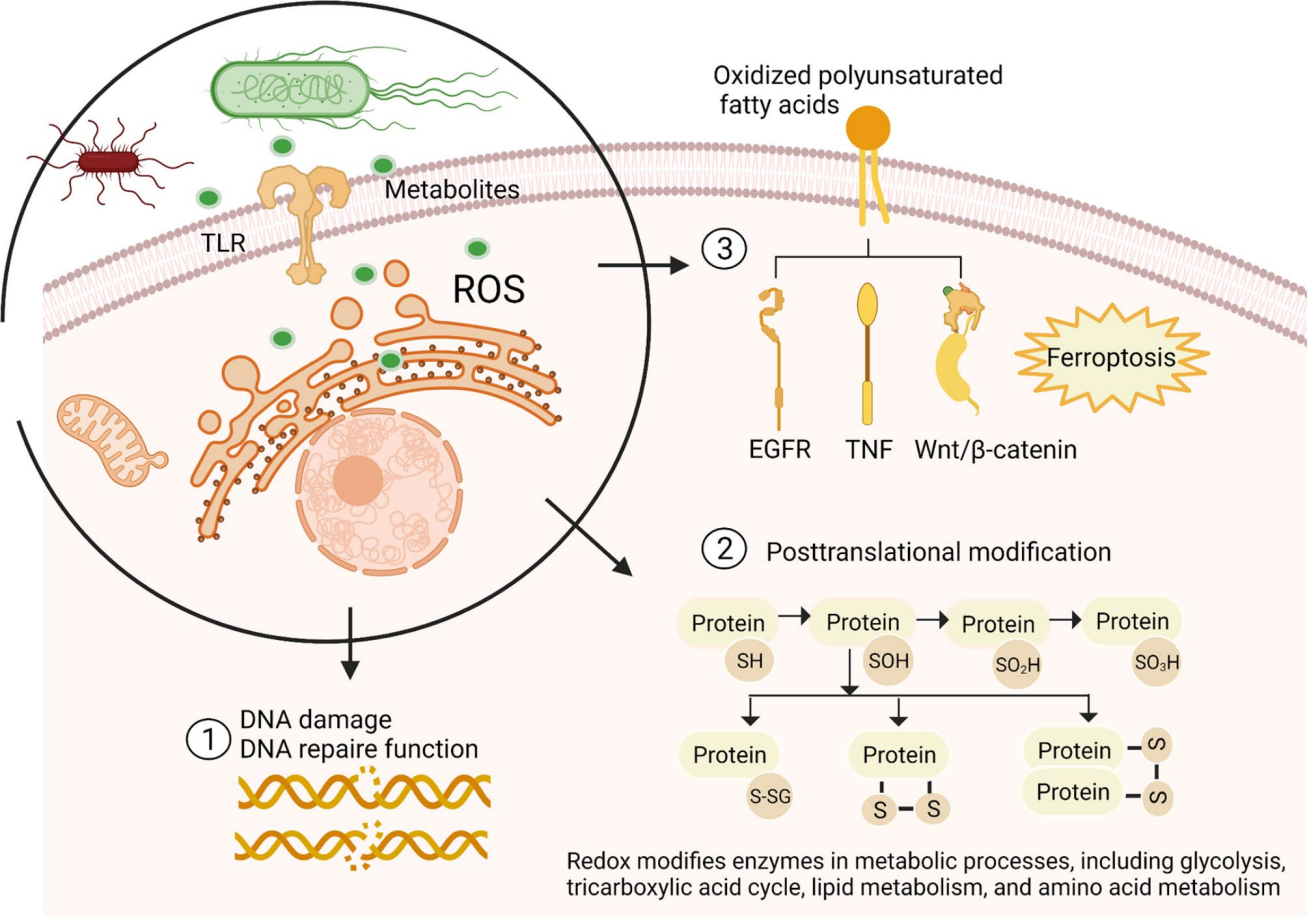
Bacteria regulated redox reactions in gastrointestinal epithelial/cancer cells. Bacteria link to gastrointestinal epithelial/cancer cells through their receptors or secreting metabolites, affecting the release of ROS from gastrointestinal epithelial/cancer cells. The overproduction of ROS can lead to DNA single strand breaks, DNA double strand breaks, DNA recombination repair system damage, and DNA mutations. Excess ROS mainly act on the sulfhydryl (RSH) side chain of the active cysteine of the protein (metabolism-related enzyme). In response to ROS, the reduced sulfhydryl groups on cysteine deprotonate and are oxidized to form sulfenic acids (RSOH). Subsequently, sulfonic acid can react with o-sulfhydryl groups to form intramolecular or intermolecular disulfide bonds (RS-SR or RS-SR’) or bind to glutathionylation (RS-SG) of glutathione (GSH). Sulfonic acid can also be further oxidized to form sulfonic acids (RSO_2_H) or sulfonic acids (RSO_3_H). The modified protein usually loses activity or function. Excess ROS react directly with polyunsaturated fatty acids on the cell membrane, causing cell membrane protein damage and influencing signal receptors on the cell membrane and various downstream signaling pathways, including the EGFR, TNF, and Wnt signaling pathways

Excess ROS-mediated destruction of DNA, proteins and lipids is shown in Fig. 3.

## Microbial metabolites are involved in cancer cell redox reactions

Bacteria can interact with GI cancer cells by secreting metabolites, including short-chain fatty acids (SCFAs), sulfides, gas molecules and even specific metabolites.

### SCFAs compete against oxidative stress in cancer cells

Colonic bacteria such as *Clostridium butyricum* and *Lactobacillus* can produce SCFAs, which mainly include acetate, butyrate acid, lactate, and propionate acid. The health benefits of SCFAs are related to their ability to regulate gene expression [72–75]. For example, *Escherichia coli* can mediate the protective effect of SCFAs on oxidative stress through the KEAP1-NRF2 signaling pathway. Under static conditions, NRF2 is blocked by its cytoplasmic inhibitor KEAP1 (Kelch-like Ech-associated protein 1). However, under oxidation and electrophilic stress, NRF2 can be synthesized and accumulate in the nucleus. NRF2 binds small Maf protein to antioxidant response elements (ARE) in target gene promoters and upregulates the expression of phase II enzymes and antioxidant enzymes to combat oxidative stress [76]. The epigenetic regulation of butyrate-induced NRF2 nuclear translocation is the main mechanism of antioxidant action [77]. Inhibitor for the apoptosis-stimulating protein of p53 (IASPP) in cancer cells can improve the stability of the NRF2 protein and promote the nuclear translocation of NRF2 by competitively binding the main inhibitory factor Keap1 of NRF2 with the DLT amino acid sequence (motifs) located at its N-terminus. NRF2 transcriptional activation of its downstream antioxidant target genes (such as NQO1, HMOX1 and FTH1) plays a role in ROS inhibition [78]. Moreover, Pant K et al. reported that butyrate could induce ROS-mediated apoptosis by modulating the miR-22/SIRT-1 pathway in hepatic cancer cells [79]. Inoue T et al. reported that butyrate could mediate ROS-related apoptosis through a complex signaling feedback loop involving p21, ROS, and p53 [80, 81]. Schlörmann W et al. reported that butyrate could mediate ROS-related proliferation and apoptosis by regulating antioxidant-relevant proteins (Cyclin D2, p21, PARP, Bid, GPx2) in colon cancer cells [82].

### Gas molecules are involved in cancer cell antioxidative stress

Sulfur-metabolizing microbes, including *Bilophila wadsworthia*, *Fusobacterium nucleatum*, and *Desulfovibrio*, have the capacity to metabolize organic compounds for energy and reduce dietary sulfur to hydrogen sulfide (H_2_S) [83, 84]. The abundance of sulfur-reducing bacteria in the colonic mucosa of patients with CRC, such as *Bilophila wadsworthia* and *Pyramidobacter*, was higher than that of healthy individuLA [85, 86]. H_2_S is mainly synthesized by L-cysteine and L-homocysteine through cystathionine-B-synthasecbs, 3-mercaptopyruvate thiotransferase (3-MST) and cysteine-Y-lyase (CTH/CSE). At 37 °C and pH 7.4, excess H_2_S molecules can dissolve in water and decompose into H^+^, HS^−^ and S^2−^ ions. HS^−^ has strong single-electron chemical inertia and has considerable scavenging capacity for ROS. Excessive H_2_S production may inhibit carcinogenesis through several mechanisms, including the Wnt signaling pathway, microRNA regulation and cancer metabolism. For instance, excess H_2_S can upregulate the expression of miR-200b and miR-22. Wnt1 was found to be a target of these two miRNAs, and a synergistic effect between miR-200b and miR-22 in the inhibition of gastric cancer growth was reported [87–89].

*Nitrobacter* and *Nitrosomonas* can produce NO in the gut [90]. The interaction between O_2_ and NO leads to the formation of peroxynitrite (ONOO). The increased reactivity of peroxynitrite leads to the production of various other NO-derived factors, called reactive nitrogen species (RNS), including reactive radical compounds nitrogen dioxide (NO_2_), hydroxyl radical (HO), and nonfree radical dinitrogen trioxide (N_2_O_3_). ONOO, together with RNS, is responsible for protein tyrosine residue nitrosation, mitochondrial energy consumption, and the induction of DNA strand breaks [91–93]. NO in the submicromolar range (< 1 μm) can reversibly inhibit cytochrome-c oxidase. It may transiently increase the leakage of superoxide from the electron transport chain. Then, superoxide can react with NO to generate peroxynitrite, which inactivates the iron/sulfur centers in cancer cell mitochondria and causes irreversible injury to the mitochondria [94]. Nitrite (NO_2_^−^) and nitrate can transfer an (NO) + moiety to exocyclic amino groups of DNA bases (purine nucleosides and nucleotides). N-nitro tyrosine residues produce uracil. Removal of uracil by uracil glycosylase without restoration of cytosine leaves an abasic site. A lesion is commonly misrepaired by insertion of adenine opposite the site during replication. Misrepair can produce a G:C-A:T transition. Hence, nitrification and deamination may lead to genetic changes in living cells [95]. High concentrations of exogenous NO are mainly toxic to cells and microorganisms, which is reflected in the inhibition of bacterial growth and the regulation of biofilm formation [96]. During cancer chemotherapy, NO can accumulate in cancer cells, resulting in cytotoxic and anticancer effects [97].

Other gaseous molecules, such as H_2_O_2_ [98, 99] and H_2_ [100, 101], are additional metabolites of microorganisms that may play important roles in multiple systems throughout the body.

### Specific metabolites are involved in regulating cancer cells

*Lactobacillus reuteri* and its oxidation metabolite reuterin inhibit ribosomal biogenesis and downstream protein translation in SW480 cell lines, thereby inhibiting cancer growth in vitro [102]. *Lactobacillus reuteri* is a natural colonizer of the human gut, and reuterin is an intermediate product of the conversion of glycerol to 1,3-propanediol [103]. Reuterin is a highly selective electrophilic substance that can interact with cysteine, resulting in irreversible oxidation, loss of protein function, and cell death [104]. Sodium sulfide protects colorectal cancer cells from reuterin-induced growth inhibition by binding to cysteine, resulting in protective and reversible oversulfidation that prevents protein oxidation. β-Galactosidase secreted by *Streptococcus thermophilus* can inhibit cell proliferation, reduce colony formation, induce cell cycle arrest, promote apoptosis of cultured CRC cells and retard the growth of CRC xenografts. The β-galactosidase-dependent production of galactose interferes with energy homeostasis to activate oxidative phosphorylation and downregulate Hippo pathway kinases, partially mediating the anticancer effects of *S. thermophilus* [105]. *Lactobacillus brevis* can produce NADPH oxidase (NOX) in an aerobic environment [106]. ROS produced by NOX inactivate nucleoredoxin, thus releasing nucleoredoxin-dependent Wnt-β-catenin signal inhibition through the separation of nucleoredoxin and dishevelled. NOX inhibits the response of cells to Wnt, including stabilizing β-catenin, expressing cyclin D1 and c-Myc through the TCF transcription factor, and accelerating cell proliferation. In colon cancer cells with a normal Wnt pathway, Nox1 mediates Wnt-induced cell growth, but not in APC-deficient colon cancer cells, which have constitutive Wnt signaling activity [107, 108].

## Application and treatment

ROS concentration is directly related to cancer cell death. Regulating ROS concentrations can be a cancer therapy stategy [109]. Based on this, synthetic biological modification of bacteria to regulate redox levels in the gut has been developed as a potential anticancer treatment. Fan et al. designed and engineered a bacterium, *Escherichia coli* MG1655, which overexpressed the NDH-2 (Type-II NADH-Menaquinone Oxidoreductase) enzyme to colonize cancer areas and increase local H_2_O production. In their study, magnetic Fe_3_O_4_ nanoparticles were covalently linked to bacteria to convert H_2_O_2_ into toxic hydroxyl radicals (•OH) for cancer treatment [110]. *Escherichia coli Nissle 1917* (EcN) is an oral probiotic that has been genetically engineered to overexpress catalase and superoxide dismutase to restore redox levels in the gut, thereby alleviating GI diseases [111]. *Onaida Shewanella* MR-1 (SO) can selectively use lactic acid instead of glucose as an energy source for respiration. During lactic acid consumption, SO can simultaneously mediate the electron transfer process that transforms reduced iron ions to bivalent iron ions. The resulting ferric ions can also be reoxidized to ferric ions by overexpressed hydrogen peroxide, thus achieving cyclic lactic acid catabolism. Using the principle of SO redox, an *Onaida Shewanella*-coupled MDH (an MOF material loaded with adriamycin) self-driven bioreactor was constructed. SO@MDH can enhance the chemotherapy effect of GI tract cancer and alleviate cancer [112–114].

Restoring ROS levels in the GI tract is a favorable way to alleviate GI diseases, including cancer. ROS nanosscavengers can be delivered to the GI tract to enhance the clearance of ROS by using the intestinal colonization ability of probiotics. In addition, a protective layer can be included on the surface of probiotics to enhance the environmental resistance and intestinal adhesion of probiotics and regulate the balance of GI microbes [115–118]. For example, the probiotic *Saccharomyces cerevisiae JKSP39* might regulate GI microbes by reducing the levels of ROS (myeloperoxidase, superoxide dismutase, catalase, H_2_O_2_, and malondialdehyde) to inhibit endoplasmic reticulum (ER) stress [115]. Hydrogel-coated *Lactobacillus reuteri* could partially remove ROS from the GI tract and protect colon HT-29 cells from oxidative damage [116]. EcN can restore ROS levels in the GI tract, enhance the abundance and diversity of GI microbes, and prevent and treat CRC-related diseases such as colitis. The retention time of EcN in the GI tract can be prolonged by coupling hyaluronic acid-polypropylene sulfide nanoparticles (HPNs) to the surface of modified EcN using a polymer of hyaluronic acid-polypropylene sulfide (HA-PPS) and encapsulating EcN cells with a polynorepinephrine layer. The prolonged presence of HPN-NE-EcN in the gut assists the ROS scavenging ability of EcN, thereby alleviating GI diseases [117].

## Conclusion

A large number of studies have shown that GI microbes such as *Escherichia coli* and *Helicobacter pylori* play an important role in the development of GI cancer. GI bacteria participate in the regulation of redox potential in the GI tract through the electronic respiratory chain and the release of metabolites. GI bacteria dynamically regulate their redox reaction according to the redox potential in the GI tract. GI bacteria can also regulate the redox potential of cancer cells by affecting the production of oxidants and antioxidants in GI cancer cells. Once oxidants (such as ROS) from the GI tract or cancer cells are present, the redox balance and homeostasis of cancer cells are destroyed. This imbalance causes redox-mediated modifications of various cellular components, such as DNA, lipids and proteins that then change the function of cancer cells. However, GI bacteria can also combat oxidative stress in cancer cells by releasing substances such as short-chain fatty acids, NO, and specific metabolites such as Reuterian proteins and NADPH oxidase.

In conclusion, GI microbes play an important role in the regulation of GI cancer-related redox levels. Using GI microbes to reduce ROS levels in the GI tract may be a beneficial pathway for alleviating GI diseases, including cancer.

## Data Availability

Not applicable.

## References

[1] Sung H, Ferlay J, Siegel RL, et al. Global Cancer Statistics 2020: GLOBOCAN estimates of incidence and Mortality Worldwide for 36 cancers in 185 countries. CA Cancer J Clin. 2021 May;71(3):209–49. 10.3322/caac.21660 33538338

[2] Sekirov I, Russell SL, Antunes LC, et al. Gut microbiota in health and disease. Physiol Rev. 2010 Jul;90(3):859–904. 10.1152/physrev.00045.2009 20664075

[3] Helmink BA, Khan MAW, Hermann A, et al. The microbiome, cancer, and cancer therapy. Nat Med. 2019 Mar;25(3):377–88. 10.1038/s41591-019-0377-7 30842679

[4] Garrett WS. Cancer and the microbiota. Science. 2015 Apr 3;348(6230):80 – 6. 10.1126/science.aaa4972 PMC5535753 25838377

[5] Tsilimigras MC, Fodor A, Jobin C. Carcinogenesis and therapeutics: the microbiota perspective. Nat Microbiol. 2017 Feb;22:2:17008. 10.1038/nmicrobiol.2017.8 PMC6423540 28225000

[6] Amieva M, Peek RM Jr. Pathobiology of Helicobacter pylori-Induced Gastric Cancer. Gastroenterology. 2016 Jan;150(1):64–78. 10.1053/j.gastro.2015.09.004 PMC4691563 26385073

[7] Fox JG, Wang TC. Inflammation, atrophy, and gastric cancer. J Clin Invest 2007 Jan;117(1):60–9. 10.1172/JCI30111 PMC1716216 17200707

[8] Nesić D, Hsu Y, Stebbins CE. Assembly and function of a bacterial genotoxin. Nat 2004 May 27;429(6990):429–33. 10.1038/nature02532 15164065

[9] Huycke MM, Abrams V, Moore DR (2002). Enterococcus faecalis produces extracellular superoxide and hydrogen peroxide that damages colonic epithelial cell DNA. Carcinogenesis.

[10] Wang X, Allen TD, May RJ (2008). Enterococcus faecalis induces aneuploidy and tetraploidy in colonic epithelial cells through a bystander effect. Cancer Res.

[11] Sun J, Kato I. Gut microbiota, inflammation and colorectal cancer. Genes Dis. 2016 Jun;3(2):130–43. 10.1016/j.gendis.2016.03.004 PMC5221561 28078319

[12] Bijnens K, Jaenen V, Wouters A et al. A Spatiotemporal Characterisation of Redox Molecules in Planarians, with a Focus on the Role of Glutathione during Regeneration. Biomolecules. 2021 May 11;11(5):714. 10.3390/biom11050714 PMC8150688 34064618

[13] Horspool AM, Chang HC. Neuron-specific regulation of superoxide dismutase amid pathogen-induced gut dysbiosis. Redox Biol. 2018 Jul;17:377–85. 10.1016/j.redox.2018.05.007 PMC6007053 29857312

[14] Campbell EL, Colgan SP. Control and dysregulation of redox signalling in the gastrointestinal tract. Nat Rev Gastroenterol Hepatol. 2019 Feb;16(2):106–20. 10.1038/s41575-018-0079-5 PMC7919748 30443019

[15] Xu CC, Yang SF, Zhu LH, et al. Regulation of N-acetyl cysteine on gut redox status and major microbiota in weaned piglets. J Anim Sci. 2014 Apr;92(4):1504–11. 10.2527/jas.2013-6755 24496840

[16] Bayir H. Reactive oxygen species. Crit Care Med. 2005 Dec;33(12 Suppl):498–501. 10.1097/01.ccm.0000186787.64500.12 16340433

[17] Hancock JT. Considerations of the importance of redox state for reactive nitrogen species action. J Exp Bot 2019 Aug 29;70(17):4323–31. 10.1093/jxb/erz067 30793204

[18] Olson KR. Are reactive Sulfur Species the new reactive oxygen species? Antioxid Redox Signal. 2020 Dec 1;33(16):1125–42. 10.1089/ars.2020.8132 32586118

[19] Li ZY, Wang L, Liu YL et al. Overlooked enhancement of chloride ion on the transformation of reactive species in peroxymonosulfate/Fe(II)/NH2OH system. Water Res. 2021 May 1;195:116973. 10.1016/j.watres.2021.116973 33677242

[20] Gao P, Pan W, Li N et al. Fluorescent probes for organelle-targeted bioactive species imaging. Chem Sci 2019 May 24;10(24):6035–71. 10.1039/c9sc01652j PMC6585876 31360411

[21] Robles-Rengel R, Florencio FJ, Muro-Pastor MI. Redox interference in nitrogen status via oxidative stress is mediated by 2-oxoglutarate in cyanobacteria. New Phytol. 2019 Oct;224(1):216–28. 10.1111/nph.15979 31168850

[22] Giles GI, Nasim MJ, Ali W et al. The Reactive Sulfur Species Concept: 15 Years On. Antioxidants (Basel). 2017 May 23;6(2):38. 10.3390/antiox6020038 PMC5488018 28545257

[23] Sannasimuthu A, Sharma D, Paray BA, et al. Intracellular oxidative damage due to antibiotics on gut bacteria reduced by glutathione oxidoreductase-derived antioxidant molecule GM15. Arch Microbiol. 2020 Jul;202(5):1127–33. 10.1007/s00203-020-01825-y 32060599

[24] Skulachev VP (2012). Mitochondria-targeted antioxidants as promising drugs for treatment of age-related brain diseases. J Alzheimers Dis.

[25] Griguer CE, Oliva CR, Kelley EE et al. Xanthine oxidase-dependent regulation of hypoxia-inducible factor in cancer cells. Cancer Res 2006 Feb 15;66(4):2257–63. 10.1158/0008-5472.CAN-05-3364 16489029

[26] Nanduri J, Vaddi DR, Khan SA, et al. Xanthine oxidase mediates hypoxia-inducible factor-2α degradation by intermittent hypoxia. PLoS ONE. 2013 Oct;4(10):e75838. 10.1371/journal.pone.0075838 PMC3790816 24124516

[27] Biasi F, Leonarduzzi G, Oteiza PI et al. Inflammatory bowel disease: mechanisms, redox considerations, and therapeutic targets. Antioxid Redox Signal 2013 Nov 10;19(14):1711–47. 10.1089/ars.2012.4530 PMC3809610 23305298

[28] Aguiar PH, Furtado C, Repolês BM, et al. Oxidative stress and DNA lesions: the role of 8-oxoguanine lesions in Trypanosoma cruzi cell viability. PLoS Negl Trop Dis. 2013 Jun;13(6):e2279. 10.1371/journal.pntd.0002279 PMC3681716 23785540

[29] Spickett CM, Pitt AR. Modification of proteins by reactive lipid oxidation products and biochemical effects of lipoxidation. Essays Biochem 2020 Feb 17;64(1):19–31. 10.1042/EBC20190058 31867621

[30] Boese AC, Kang S. Mitochondrial metabolism-mediated redox regulation in cancer progression. Redox Biol. 2021 Jun;42:101870. 10.1016/j.redox.2021.101870 PMC8113029 33509708

[31] González-Flecha B, Demple B. Metabolic sources of hydrogen peroxide in aerobically growing Escherichia coli. J Biol Chem 1995 Jun 9;270(23):13681–7. 10.1074/jbc.270.23.13681 7775420

[32] Nothling MD, Cao H, McKenzie TG et al. Bacterial redox potential Powers Controlled Radical polymerization. J Am Chem Soc 2021 Jan 13;143(1):286–93. 10.1021/jacs.0c10673 33373526

[33] Xu J, Xu C, Chen X, et al. Regulation of an antioxidant blend on intestinal redox status and major microbiota in early weaned piglets. Nutrition. 2014 May;30(5):584–9. 10.1016/j.nut.2013.10.018 24698350

[34] Camerini S, Marcocci L, Picarazzi L et al. Type E Botulinum Neurotoxin-Producing Clostridium butyricum strains are aerotolerant during vegetative growth. mSystems. 2019 Apr 30;4(2):e00299–18. 10.1128/mSystems.00299-18 PMC6495232 31058231

[35] Zhou JJ, Shen JT, Wang XL, et al. Metabolism, morphology and transcriptome analysis of oscillatory behavior of Clostridium butyricum during long-term continuous fermentation for 1,3-propanediol production. Biotechnol Biofuels. 2020 Nov;25(1):191. 10.1186/s13068-020-01831-8 PMC7690194 33292405

[36] Szczepanowski P, Noszka M, Żyła-Uklejewicz D, et al. HP1021 is a redox switch protein identified in Helicobacter pylori. Nucleic Acids Res. 2021 Jul;9(12):6863–79. 10.1093/nar/gkab440 PMC8266642 34139017

[37] Keum N, Giovannucci EL. Folic acid fortification and colorectal cancer risk. Am J Prev Med. 2014 Mar;46(3 Suppl 1):65–72. 10.1016/j.amepre.2013.10.025 24512932

[38] Jose S, Bhalla P, Suraishkumar GK. Oxidative stress decreases the redox ratio and folate content in the gut microbe, Enterococcus durans (MTCC 3031). Sci Rep. 2018 Aug;14(1):12138. 10.1038/s41598-018-30691-4 PMC6092354 30108274

[39] Sies H, Jones DP. Reactive oxygen species (ROS) as pleiotropic physiological signaling agents. Nat Rev Mol Cell Biol. 2020 Jul;21(7):363–83. 10.1038/s41580-020-0230-3 32231263

[40] Grasberger H, El-Zaatari M, Dang DT, et al. Dual oxidases control release of hydrogen peroxide by the gastric epithelium to prevent Helicobacter felis infection and inflammation in mice. Gastroenterology. 2013 Nov;145(5):1045–54. 10.1053/j.gastro.2013.07.011 PMC3805753 23860501

[41] Wen J, Wang Y, Gao C, et al. Helicobacter pylori infection promotes aquaporin 3 expression via the ROS-HIF-1α-AQP3-ROS loop in stomach mucosa: a potential novel mechanism for cancer pathogenesis. Oncogene. 2018 Jun;37(26):3549–61. 10.1038/s41388-018-0208-1 29563612

[42] Lesiów MK, Komarnicka UK, Kyzioł A, et al. ROS-mediated lipid peroxidation as a result of Cu(ii) interaction with FomA protein fragments of F. nucleatum: relevance to colorectal carcinogenesis. Metallomics. 2019 Dec;11(12):2066–77. 10.1039/c9mt00179d 31657425

[43] Lyons NJ, Giri R, Begun J, Clark D, Proud D, He Y, Hooper JD, Kryza T. Reactive oxygen species as mediators of Disease Progression and Therapeutic Response in Colorectal Cancer. Antioxid Redox Signal. 2023 Apr 25. 10.1089/ars.2022.0127 36792932

[44] Nie S, Wang A, Yuan Y. Comparison of clinicopathological parameters, prognosis, micro-ecological environment and metabolic function of Gastric Cancer with or without Fusobacterium sp. Infection. J Cancer. 2021 Jan 1;12(4):1023–1032. 10.7150/jca.50918 PMC7797643 33442401

[45] Wang Y, Li H, Li T, et al. Cytoprotective effect of Streptococcus thermophilus against oxidative stress mediated by a novel peroxidase (EfeB). J Dairy Sci. 2018 Aug;101(8):6955–63. 10.3168/jds.2018-14601 29803415

[46] Zhang C, Xin Y, Wang Y et al. Identification of a novel dye-decolorizing peroxidase, EfeB, translocated by a twin-arginine translocation system in Streptococcus thermophilus CGMCC 7.179. Appl Environ Microbiol. 2015 Sep;81(18):6108–19. 10.1128/AEM.01300-15 PMC4542251 26092460

[47] Strickertsson JA, Desler C, Martin-Bertelsen T, et al. Enterococcus faecalis infection causes inflammation, intracellular oxphos-independent ROS production, and DNA damage in human gastric cancer cells. PLoS ONE. 2013 Apr;30(4):e63147. 10.1371/journal.pone.0063147 PMC3639970 23646188

[48] Davalli P, Marverti G, Lauriola A, et al. Targeting Oxidatively Induced DNA damage response in Cancer: Opportunities for Novel Cancer Therapies. Oxid Med Cell Longev. 2018 Mar;2018:27. 10.1155/2018/2389523 PMC5892224 29770165

[49] Kaneko K, Akuta T, Sawa T (2008). Mutagenicity of 8-nitroguanosine, a product of nitrative nucleoside modification by reactive nitrogen oxides, in mammalian cells. Cancer Lett.

[50] Park SH, Kim Y, Ra JS, et al. Timely termination of repair DNA synthesis by ATAD5 is important in oxidative DNA damage-induced single-strand break repair. Nucleic Acids Res. 2021 Nov;18(20):11746–64. 10.1093/nar/gkab999 PMC8599757 34718749

[51] Jamsen JA, Sassa A, Perera L et al. Structural basis for proficient oxidized ribonucleotide insertion in double strand break repair. Nat Commun 2021 Aug 20;12(1):5055. 10.1038/s41467-021-24486-x PMC8379156 34417448

[52] Cannan WJ, Tsang BP, Wallace SS (2014). Nucleosomes suppress the formation of double-strand DNA breaks during attempted base excision repair of clustered oxidative damages. J Biol Chem.

[53] Wang S, Chen Z, Zhu S, et al. PRDX2 protects against oxidative stress induced by H. pylori and promotes resistance to cisplatin in gastric cancer. Redox Biol. 2020 Jan;28:101319. 10.1016/j.redox.2019.101319 PMC6811995 31536951

[54] Salzano S, Checconi P, Hanschmann EM et al. Linkage of inflammation and oxidative stress via release of glutathionylated peroxiredoxin-2, which acts as a danger signal. Proc Natl Acad Sci U S A. 2014 Aug 19;111(33):12157-62. 10.1073/pnas.1401712111 PMC4143057 25097261

[55] Somyajit K, Gupta R, Sedlackova H et al. Redox-sensitive alteration of replisome architecture safeguards genome integrity. Sci 2017 Nov 10;358(6364):797–802. 10.1126/science.aao3172 29123070

[56] Sedletska Y, Radicella JP, Sage E (2013). Replication fork collapse is a major cause of the high mutation frequency at three-base lesion clusters. Nucleic Acids Res.

[57] Degtyareva NP, Heyburn L, Sterling J, et al. Oxidative stress-induced mutagenesis in single-strand DNA occurs primarily at cytosines and is DNA polymerase zeta-dependent only for adenines and guanines. Nucleic Acids Res. 2013 Oct;41(19):8995–9005. 10.1093/nar/gkt671 PMC3799438 23925127

[58] Goodwin AC, Destefano Shields CE, Wu S et al. Polyamine catabolism contributes to enterotoxigenic Bacteroides fragilis-induced colon tumorigenesis. Proc Natl Acad Sci U S A 2011 Sep 13;108(37):15354–9. 10.1073/pnas.1010203108 PMC3174648 21876161

[59] Tan J, Duan M, Yadav T et al. An R-loop-initiated CSB-RAD52-POLD3 pathway suppresses ROS-induced telomeric DNA breaks. Nucleic Acids Res 2020 Feb 20;48(3):1285–300. 10.1093/nar/gkz1114 PMC7026659 31777915

[60] Meng J, Fu L, Liu K et al. Global profiling of distinct cysteine redox forms reveals wide-ranging redox regulation in C. elegans. Nat Commun 2021 Mar 3;12(1):1415. 10.1038/s41467-021-21686-3 PMC7930113 33658510

[61] Moldogazieva NT, Lutsenko SV, Terentiev AA. Reactive oxygen and Nitrogen Species-Induced protein modifications: implication in carcinogenesis and anticancer therapy. Cancer Res 2018 Nov 1;78(21):6040–7. 10.1158/0008-5472.CAN-18-0980 30327380

[62] Filipovic MR, Zivanovic J, Alvarez B et al. Chemical Biology of H2S Signaling through Persulfidation. Chem Rev 2018 Feb 14;118(3):1253–337. 10.1021/acs.chemrev.7b00205 PMC6029264 29112440

[63] Song IK, Lee JJ, Cho JH et al. Degradation of Redox-Sensitive Proteins including peroxiredoxins and DJ-1 is promoted by oxidation-induced conformational changes and ubiquitination. Sci Rep 2016 Oct 5;6:34432. 10.1038/srep34432 PMC5050490 27703196

[64] Smith KA, Waypa GB, Schumacker PT. Redox signaling during hypoxia in mammalian cells. Redox Biol. 2017 Oct;13:228–34. 10.1016/j.redox.2017.05.020 PMC5460738 28595160

[65] Abd-El-Raouf R, Ouf SA, Gabr MM et al. Escherichia coli foster bladder cancer cell line progression via epithelial mesenchymal transition, stemness and metabolic reprogramming. Sci Rep. 2020 Oct 22;10(1):18024. 10.1038/s41598-020-74390-5 PMC7581527 33093503

[66] Hurd TR, Collins Y, Abakumova I et al. Inactivation of pyruvate dehydrogenase kinase 2 by mitochondrial reactive oxygen species. J Biol Chem. 2012 Oct 12;287(42):35153–35160. 10.1074/jbc.M112.400002 PMC3471752 22910903

[67] Humphries KM, Szweda LI. Selective inactivation of alpha-ketoglutarate dehydrogenase and pyruvate dehydrogenase: reaction of lipoic acid with 4-hydroxy-2-nonenal. Biochemistry. 1998 Nov;10(45):15835–41. 10.1021/bi981512h 9843389

[68] Ezraty B, Gennaris A, Barras F, et al. Oxidative stress, protein damage and repair in bacteria. Nat Rev Microbiol. 2017 Jul;15(7):385–96. 10.1038/nrmicro.2017.26 28420885

[69] Dodson M, Castro-Portuguez R, Zhang DD. NRF2 plays a critical role in mitigating lipid peroxidation and ferroptosis. Redox Biol. 2019 May;23:101107. 10.1016/j.redox.2019.101107 PMC6859567 30692038

[70] Ingold I, Berndt C, Schmitt S et al. Selenium utilization by GPX4 is required to Prevent Hydroperoxide-Induced ferroptosis. Cell 2018 Jan 25;172(3):409–422e21. 10.1016/j.cell.2017.11.048 29290465

[71] Nordzieke DE, Medraño-Fernandez I. The plasma membrane: a platform for intra- and intercellular Redox Signaling. Antioxidants (Basel). 2018 Nov 20;7(11):168. 10.3390/antiox7110168 PMC6262572 30463362

[72] Wang G, Yu Y, Wang YZ, et al. Role of SCFAs in gut microbiome and glycolysis for colorectal cancer therapy. J Cell Physiol. 2019 Aug;234(10):17023–49. 10.1002/jcp.28436 30888065

[73] Houghton CA, Fassett RG, Coombes JS (2016). Sulforaphane and other Nutrigenomic Nrf2 activators: can the Clinician’s expectation be matched by the reality?. Oxid Med Cell Longev.

[74] Afrin S, Giampieri F, Gasparrini M et al. Dietary phytochemicals in colorectal cancer prevention and treatment: a focus on the molecular mechanisms involved. Biotechnol Adv 2020 Jan-Feb;38:107322. 10.1016/j.biotechadv.2018.11.011 30476540

[75] Chikara S, Nagaprashantha LD, Singhal J et al. Oxidative stress and dietary phytochemicals: role in cancer chemoprevention and treatment. Cancer Lett 2018 Jan 28;413:122–34. 10.1016/j.canlet.2017.11.002 29113871

[76] Itoh K, Wakabayashi N, Katoh Y et al. Keap1 represses nuclear activation of antioxidant responsive elements by Nrf2 through binding to the amino-terminal Neh2 domain. Genes Dev. 1999 Jan 1;13(1):76–86. 10.1101/gad.13.1.76 PMC316370 9887101

[77] González-Bosch C, Boorman E, Zunszain PA, et al. Short-chain fatty acids as modulators of redox signaling in health and disease. Redox Biol. 2021 Nov;47:102165. 10.1016/j.redox.2021.102165 PMC8577496 34662811

[78] Ge W, Zhao K, Wang X et al. iASPP is an antioxidative factor and drives Cancer Growth and Drug Resistance by competing with Nrf2 for Keap1 binding. Cancer Cell 2017 Nov 13;32(5):561–573e6. 10.1016/j.ccell.2017.09.008 29033244

[79] Pant K, Yadav AK, Gupta P, et al. Butyrate induces ROS-mediated apoptosis by modulating miR-22/SIRT-1 pathway in hepatic cancer cells. Redox Biol. 2017 Aug;12:340–9. 10.1016/j.redox.2017.03.006 PMC5350572 28288414

[80] Inoue T, Kato K, Kato H, et al. Level of reactive oxygen species induced by p21Waf1/CIP1 is critical for the determination of cell fate. Cancer Sci. 2009 Jul;100(7):1275–83. 10.1111/j.1349-7006.2009.01166.x PMC11158913 19432898

[81] Macip S, Igarashi M, Fang L et al. Inhibition of p21-mediated ROS accumulation can rescue p21-induced senescence. EMBO J. 2002 May 1;21(9):2180-8. 10.1093/emboj/21.9.2180 PMC125979 11980715

[82] Schlörmann W, Horlebein C, Hübner SM et al. Potential Role of ROS in Butyrate- and Dietary Fiber-Mediated Growth Inhibition and Modulation of Cell Cycle-, Apoptosis- and Antioxidant-Relevant Proteins in LT97 Colon Adenoma and HT29 Colon Carcinoma Cells. Cancers (Basel). 2023 Jan 10;15(2):440. 10.3390/cancers15020440 PMC9857069 36672389

[83] Ramasamy S, Singh S, Taniere P, et al. Sulfide-detoxifying enzymes in the human colon are decreased in cancer and upregulated in differentiation. Am J Physiol Gastrointest Liver Physiol. 2006 Aug;291(2):G288–96. 10.1152/ajpgi.00324.2005 16500920

[84] Magee EA, Richardson CJ, Hughes R, et al. Contribution of dietary protein to sulfide production in the large intestine: an in vitro and a controlled feeding study in humans. Am J Clin Nutr. 2000 Dec;72(6):1488–94. 10.1093/ajcn/72.6.1488 11101476

[85] Yazici C, Wolf PG, Kim H, et al. Race-dependent association of sulfidogenic bacteria with colorectal cancer. Gut. 2017 Nov;66(11):1983–94. 10.1136/gutjnl-2016-313321 PMC5575988 28153960

[86] Nguyen LH, Cao Y, Hur J, et al. The Sulfur Microbial Diet is Associated with increased risk of early-onset Colorectal Cancer Precursors. Gastroenterology. 2021 Nov;161(5):1423–1432e4. 10.1053/j.gastro.2021.07.008 PMC8545755 34273347

[87] Attene-Ramos MS, Wagner ED, Gaskins HR et al. Hydrogen sulfide induces direct radical-associated DNA damage. Mol Cancer Res 2007 May;5(5):455–9. 10.1158/1541-7786.MCR-06-0439 17475672

[88] Tang H, Kong Y, Guo J, et al. Diallyl disulfide suppresses proliferation and induces apoptosis in human gastric cancer through Wnt-1 signaling pathway by up-regulation of miR-200b and miR-22. Cancer Lett. 2013 Oct;28(1):72–81. 10.1016/j.canlet.2013.06.027 23851184

[89] Lee ZW, Teo XY, Tay EY, et al. Utilizing hydrogen sulfide as a novel anti-cancer agent by targeting cancer glycolysis and pH imbalance. Br J Pharmacol. 2014 Sep;171(18):4322–36. 10.1111/bph.12773 PMC4241097 24827113

[90] Salimian Rizi B, Achreja A, Nagrath D. Nitric oxide: the Forgotten child of Tumor Metabolism. Trends Cancer. 2017 Sep;3(9):659–72. 10.1016/j.trecan.2017.07.005 PMC5679229 28867169

[91] Grimm EA, Sikora AG, Ekmekcioglu S. Molecular pathways: inflammation-associated nitric-oxide production as a cancer-supporting redox mechanism and a potential therapeutic target. Clin Cancer Res 2013 Oct 15;19(20):5557–63. 10.1158/1078-0432.CCR-12-1554 PMC3873873 23868870

[92] Hu Y, Lv T, Ma Y et al. Nanoscale Coordination Polymers for synergistic NO and chemodynamic therapy of Liver Cancer. Nano Lett 2019 Apr 10;19(4):2731–8. 10.1021/acs.nanolett.9b01093 30919635

[93] Pacher P, Beckman JS, Liaudet L. Nitric oxide and peroxynitrite in health and disease. Physiol Rev. 2007 Jan;87(1):315–424. 10.1152/physrev.00029.2006 PMC2248324 17237348

[94] Ferrer-Sueta G, Campolo N, Trujillo M et al. Biochemistry of Peroxynitrite and Protein Tyrosine Nitration. Chem Rev 2018 Feb 14;118(3):1338–408. 10.1021/acs.chemrev.7b00568 29400454

[95] Wink DA, Kasprzak KS, Maragos CM et al. DNA deaminating ability and genotoxicity of nitric oxide and its progenitors. Sci 1991 Nov 15;254(5034):1001–3. 10.1126/science.1948068 1948068

[96] Yuan Z, Lin C, He Y, et al. Near-Infrared light-triggered nitric-oxide-enhanced photodynamic therapy and low-temperature Photothermal Therapy for Biofilm Elimination. ACS Nano. 2020 Mar;24(3):3546–62. 10.1021/acsnano.9b09871 32069025

[97] Kim J, Yung BC, Kim WJ et al. Combination of nitric oxide and drug delivery systems: tools for overcoming drug resistance in chemotherapy. J Control Release 2017 Oct 10;263:223–30. 10.1016/j.jconrel.2016.12.026 PMC5484762 28034787

[98] Crowley SM, Vallance BA. Microbial respiration in the Colon: using H2O2 to catch your breath. Cell Host Microbe 2020 Dec 9;28(6):771–3. 10.1016/j.chom.2020.11.006 33301714

[99] Stone JR, Yang S. Hydrogen peroxide: a signaling messenger. Antioxid Redox Signal 2006 Mar-Apr;8(3–4):243–70. 10.1089/ars.2006.8.243 16677071

[100] Birg A, Ritz N, Barton LL, et al. Hydrogen availability is dependent on the actions of both hydrogen-producing and hydrogen-consuming microbes. Dig Dis Sci. 2023 Apr;68(4):1253–9. 10.1007/s10620-022-07743-x 36323965

[101] Yan H, Fan M, Liu H, et al. Microbial hydrogen “manufactory” for enhanced gas therapy and self-activated immunotherapy via reduced immune escape. J Nanobiotechnol. 2022 Jun;15(1):280. 10.1186/s12951-022-01440-7 PMC9199139 35705974

[102] Bell HN, Rebernick RJ, Goyert J et al. Reuterin in the healthy gut microbiome suppresses colorectal cancer growth through altering redox balance. Cancer Cell 2022 Feb 14;40(2):185–200e6. 10.1016/j.ccell.2021.12.001 PMC8847337 34951957

[103] Martín-Cabrejas I, Langa S, Gaya P, et al. Optimization of reuterin production in cheese by Lactobacillus reuteri. J Food Sci Technol. 2017 Apr;54(5):1346–9. 10.1007/s13197-017-2563-2 PMC5380638 28416886

[104] Zhang Z, Wang K, Oh JH, et al. A phylogenetic view on the role of glycerol for growth enhancement and reuterin formation in Limosilactobacillus reuteri. Front Microbiol. 2020 Dec;21:11:601422. 10.3389/fmicb.2020.601422 PMC7779471 33408707

[105] Li Q, Hu W, Liu WX et al. Streptococcus thermophilus inhibits colorectal tumorigenesis through secreting β-Galactosidase. Gastroenterol 2021 Mar;160(4):1179–1193e14. 10.1053/j.gastro.2020.09.003 32920015

[106] Goodman RP, Markhard AL, Shah H, et al. Hepatic NADH reductive stress underlies common variation in metabolic traits. Nature. 2020 Jul;583(7814):122–6. 10.1038/s41586-020-2337-2 PMC7536642 32461692

[107] Pérez S, Taléns-Visconti R, Rius-Pérez S, et al. Redox signaling in the gastrointestinal tract. Free Radic Biol Med. 2017 Mar;104:75–103. 10.1016/j.freeradbiomed.2016.12.048 28062361

[108] Kajla S, Mondol AS, Nagasawa A, et al. A crucial role for Nox 1 in redox-dependent regulation of Wnt-β-catenin signaling. FASEB J. 2012 May;26(5):2049–59. 10.1096/fj.11-196360 22278940

[109] Dharmaraja AT. Role of reactive oxygen species (ROS) in therapeutics and drug resistance in Cancer and Bacteria. J Med Chem 2017 Apr 27;60(8):3221–40. 10.1021/acs.jmedchem.6b01243 28135088

[110] Fan JX, Peng MY, Wang H, et al. Engineered Bacterial Bioreactor for Tumor Therapy via Fenton-Like reaction with localized H2 O2 generation. Adv Mater. 2019 Apr;31(16):e1808278. 10.1002/adma.201808278 30803049

[111] Zhou J, Li M, Chen Q et al. Programmable probiotics modulate inflammation and gut microbiota for inflammatory bowel disease treatment after effective oral delivery. Nat Commun 2022 Jun 14;13(1):3432. 10.1038/s41467-022-31171-0 PMC9198027 35701435

[112] Wang JW, Chen QW, Luo GF et al. A self-driven Bioreactor based on Bacterium-Metal-Organic Framework Biohybrids for Boosting Chemotherapy via Cyclic Lactate Catabolism. ACS Nano. 2021 Nov 6. 10.1021/acsnano.1c06123 34747172

[113] Chen QW, Wang JW, Wang XN et al. Inhibition of Tumor Progression through the Coupling of Bacterial Respiration with Tumor Metabolism. Angew Chem Int Ed Engl. 2020 Nov 23;59(48):21562–21570. 10.1002/anie.202002649 32779303

[114] Pinchuk GE, Rodionov DA, Yang C, et al. Genomic reconstruction of Shewanella oneidensis MR-1 metabolism reveals a previously uncharacterized machinery for lactate utilization. Proc Natl Acad Sci U S A. 2009 Feb;24(8):2874–9. 10.1073/pnas.0806798106 PMC2636740 19196979

[115] Zeng X, Li X, Yue Y et al. Ameliorative effect of Saccharomyces cerevisiae JKSP39 on Fusobacterium nucleatum and Dextran Sulfate Sodium-Induced Colitis Mouse Model. J Agric Food Chem 2022 Nov 9;70(44):14179–92. 10.1021/acs.jafc.2c05338 36260319

[116] Huang L, Wang J, Kong L et al. ROS-responsive hyaluronic acid hydrogel for targeted delivery of probiotics to relieve colitis. Int J Biol Macromol. 2022 Dec 1;222(Pt A):1476–1486. 10.1016/j.ijbiomac.2022.09.247 36195227

[117] Liu J, Wang Y, Heelan WJ et al. Mucoadhesive probiotic backpacks with ROS nanoscavengers enhance the bacteriotherapy for inflammatory bowel diseases. Sci Adv 2022 Nov 11;8(45):eabp8798. 10.1126/sciadv.abp8798 PMC9651739 36367930

[118] Nantapong N, Murata R, Trakulnaleamsai S, et al. The effect of reactive oxygen species (ROS) and ROS-scavenging enzymes, superoxide dismutase and catalase, on the thermotolerant ability of Corynebacterium glutamicum. Appl Microbiol Biotechnol. 2019 Jul;103(13):5355–66. 10.1007/s00253-019-09848-2 31041469

